# Numerical investigation of acoustic cavitation and viscoelastic tissue deformation

**DOI:** 10.1016/j.ultsonch.2024.106757

**Published:** 2024-01-09

**Authors:** Jaesung Park, Gihun Son

**Affiliations:** Department of Mechanical Engineering, Sogang University, 35 Baekbeom-ro, Mapo-gu, Seoul 04107, South Korea

**Keywords:** Acoustic cavitation, Bubble growth, Bubble collapse, Tissue deformation, Bubble-tissue interaction

## Abstract

Acoustic cavitation and tissue deformation are studied by modifying a level-set method for compressible two-phase flows to consider viscoelastic tissue deformation. The numerical simulations performed using different shear moduli and bubble-tissue distances demonstrate various interactions between bubble and viscoelastic tissue, including inverted cone-shape bubbles, bubble migration, liquid jet formation, compressive and expansive tissue deformation, and tissue perforation. The bubble is observed to grow larger with increasing tissue bulk modulus and density. The maximum tissue deformation generally increases with decreasing initial bubble-tissue distance and with increasing tissue bulk modulus and density. The tissue shear modulus conditions that maximize tissue deformation are in the range of 1–10 MPa, unless the tissue density is very large.

## Highlights

• A numerical method for acoustic cavitation and viscoelastic tissue deformation is presented.
• Tissue deformation is maximum at shear modulus in the range of 1–10 MPa.
• Tissue deformation generally increases with tissue bulk modulus and density.
• Liquid jet formation has a significant effect on large tissue deformation with perforation.

## 1 Introduction

Acoustic cavitation, which can be practically defined as the growth and subsequent collapses of pre-existing bubble nuclei by ultrasound radiation [1], [2], is a promising tool in a variety of engineering applications with including water decontamination [3], [4], surface cleaning [5], [6], and targeted drug delivery [7], [8]. The liquid jet generated during asymmetric bubble collapse mechanically destroys surrounding organic matter, including viruses, enabling high-quality wastewater treatment [4]. The microstreaming, caused by a liquid jet spreading over the surrounding biofilm, creates localized shear stresses and removes contaminants attached to the underlying substrate [6]. Oscillating microbubbles can exert mechanical force on nearby tissues, promoting local drug uptake and improving drug penetration efficacy into brain or tumor tissues [7]. Ultrasonic cleaning and drug delivery should be done while reducing substrate erosion and biological damage, and wastewater treatment should be performed to maximize inactivation of bacterial cells or viruses [6], [7]. Understanding the interactions between cavitation bubbles and viscoelastic tissues or cells is important for various applications of acoustic cavitation.

Extensive theoretical models for acoustic cavitation have been developed based on the Keller-Miksis (KM) equation including the liquid compressibility effect [2]. Yasui [9] analyzed the influence of phase change due to thermal conduction on acoustic cavitation dynamics by modifying the KM equation and using a temperature boundary layer approximation. The results considering thermal conduction were found to be in better agreement with experimental data than the results without conduction. Storey and Szeri [10] investigated cavitation bubble collapse through a comprehensive analysis including the effects of phase change, diffusion and chemical reactions. They reported that chemical reaction of vapor inside the bubble can significantly lower the bubble temperature. Hong and Son [11] developed a general numerical model by modifying the theoretical equation and determining the evaporation rate directly from the temperature gradients at the interface, and investigated the cavitation threshold by varying non-condensible bubble nucleus radius and acoustic frequency. Merouani’s group [12], [13], [14] also extended the KM model to include the phase change and chemical reaction effects. They analyzed the influences of methanol concentration and acoustic conditions on the generation of hydrogen and reactive species in methanol pyrolysis [12], [13], and analyzed the water dissolution mechanism depending on the ultrasonic frequency and number of acoustic cycle [14]. Although the theoretical models have been widely applied to understand acoustic cavitation phenomena, they have limitations in considering non-spherical bubble behavior and bubble-solid interaction.

Experimental researches on the interactions between single cavitation bubbles and nearby elastic boundaries have been performed using laser-induced or spark-induced bubble generation techniques [15], [16], [17]. Brujan et al. [15] found that a mushroom-shaped bubble appears during the bubble contraction stage due to the repulsion of the elastic boundary deformed by the laser-generated bubble. Ma et al. [17] compared the bubble dynamics using spark-induced cavitation bubbles under two different boundaries with the same elastic modulus but different thickness. However, few experimental studies have been performed under ultrasonic cavitation conditions due to the difficulties in controlling the bubble generation position and size [18], [19].

Alternatively, numerical simulations have been performed for bubble-tissue interactions under ultrasonic conditions. Fong et al. [20] analyzed the bubble growth and collapse near various biological tissues by combining a boundary element method based on inviscid and incompressible flow assumptions with a model that treats the biological tissue as an infinite fluid with elastic properties at the fluid–fluid boundary. They studied the influences of acoustic frequency and elastic modulus on the interaction between bubble and various biomaterials. Kobayashi et al. [21] numerically simulated shock–bubble interactions for various biomaterials by combining an equation of state to incorporate the compressibility of tissues and an improved ghost fluid technique for reliable calculations of compressible two-phase flows. However, their method has limitations in that it treats the biological tissue as a compressible fluid without elasticity. Guo et al. [22] studied the deformation of spherical cells depending on initial bubble generation size and location under ultrasonic conditions by modeling the elasticity of cell membrane with surface tension proportional to the surface area. The above numerical approaches to mimic biological cell or tissue have limitation in reflecting viscoelastic feature [23], [24] and accurately predicting later stage of tissue perforation [25].

More realistic simulations have been conducted by calculating tissue deformation by the finite element method (FEM) with a tissue model that regards the tissue as an elastic solid. Chahine et al. [26] investigated the effects of secondary collapse of toroidal-shape bubble and liquid jet on the cleaning of soft materials by considering the tissue as a solid with elastoplastic properties and using the FEM to calculate tissue deformation. Zevnik and Dular [27] analyzed bacterial cell damage during bubble collapse by modeling the cell as an elastic solid with multi-layered shell structures and using the FEM for cell deformation. However, only a few numerical simulations have been performed for the interactions between cavitation bubbles and viscoelastic solid tissues under ultrasonic conditions.

Full Eulerian methods [28], [29], [30], [31], which are advantageous for describing large solid deformation without mesh regeneration [32], [33], have also been applied to various biological tissues. Ii et al. [29] simulated the complex migration and deformation patterns of blood cells in practical intravascular situations by modeling the multiple red blood cells as biconcave capsules and employing the full Eulerian method. Recently, Koukas et al. [30] investigated the interactions between shock wave and bubble near biological tissues with two different shear moduli by extending the fluid tissue model to include the elastic features of solid tissues. Their computations successfully represented a variety of lithotripsy situations, neglecting the viscosity and surface tension effects which can be important in the acoustic cavitation dynamics of pre-existing nuclei or microbubbles [27], [34]. Park and Son [31] performed a preliminary study on ultrasound-driven dodecafluoropentane (DDFP) bubble motion and tissue deformation, which is applicable to medical imaging and therapy, by improving a level-set (LS) method for bubble dynamics under ultrasonic conditions to include the elastic properties of tissue using the full Eulerian method. They investigated the effects of ultrasonic parameters, bubble-tissue distances and shear moduli on bubble-tissue interactions. However, their computations were limited to only thin incompressible solid tissues, which can not take into account ultrasound transmission through the solid regions.

In this work, a more general numerical method is developed for acoustic cavitaion bubble motion near a viscoelastic tissue by extending the LS method to treat the elastic properties and acoustic impedance of tissues as well as the effects of liquid and gas compressibilities. We also perform a more comprehensive analysis of acoustic cavitation and tissue deformation by varying tissue properties, such as shear modulus, bulk modulus and density, as well as bubble-tissue distances.

## 2 Numerical analysis

**Fig. 1:**
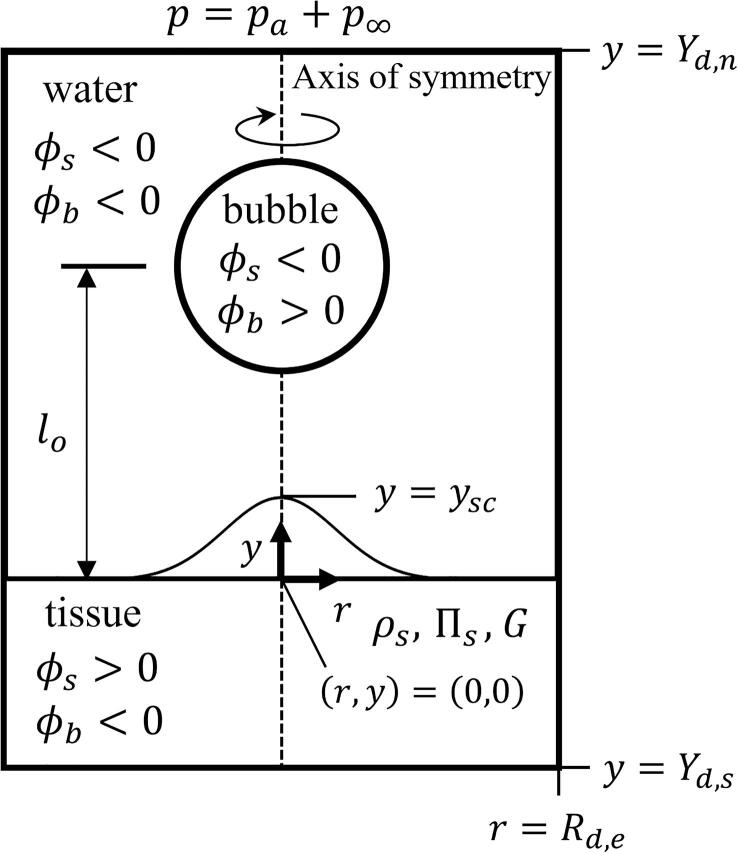
Schematic for analysis of acoustic cavitation and viscoelastic tissue deformation.

Fig. 1 shows the schematic for analysis of acoustic cavitation of a pre-existing air bubble near a viscoelastic solid tissue. Computations are conducted under the assumptions that the fluid flow and solid motion axisymmetric, and all physical properties for each phases are constant except density. The compressibility effects of water and solid are treated by evaluating the water and solid tissue pressures from the Tait equations,

(1) $$p_{w/s}=p_{\infty }+\Pi_{w/s}\left[{\left(\frac{\rho_{w/s}}{\rho_{w/s,\infty }}\right)}^{\gamma_{w/s}}-1\right]$$

where $p_{\infty }=101.3\;\mathrm{kPa}$ and $\gamma_{w}=\gamma_{s}=7.15$, regarding that the volume fraction of water is nearly 80% of the cell tissue [35]. To more accurately consider the high-pressure bubble upon collapse, the bubble pressure is determined from the van der Waals equation for isothermal conditions,

(2) $$p_{b}=(p_{\infty }+a^{*}\rho_{b,\infty }^{2})\left(\frac{\rho_{b,\infty }^{-1}-b^{*}}{\rho_{b}^{-1}-b^{*}}\right)-a^{*}\rho_{b}^{2}$$

where

(3) $$a^{*}=\frac{27}{64}\frac{R_{g}^{2}T_{c}^{2}}{p_{c}},\;\;b^{*}=\frac{R_{g}\;T_{c}}{8p_{c}}$$

Here, the subscript *c* denotes the critical state of air bubble and $R_{g}=287\;J/\mathrm{kgK}$.

### 2.1 Governing equations

The LS method described in our previous studies for various two-phase flow applications [35], [36], [37] is extended to investigate acoustic cavitation and resulting tissue deformation. The unified governing equations for bubble, water and tissue regions are expressed as

(4) $$\frac{\partial \rho }{\partial t}+\nabla \cdot (u\rho )=0$$

(5) $$\frac{\partial \rho u}{\partial t}+\nabla \cdot (u\rho u)=-(\nabla p+\sigma \kappa \nabla H_{b})+\nabla \cdot \left[\mu \left(\nabla u+\nabla u^{T}-\frac{2}{3}(\nabla \cdot u)I\right)+\alpha_{s}\tau_{e}\right]$$

(6) $$\frac{\partial B}{\partial t}+u\cdot \nabla B=(\nabla u^{T})\cdot B+B\cdot (\nabla u)$$

where

(7) $$\tau_{e}=G(B-I)$$

(8) $$\rho =\left[\rho_{b}\alpha_{b}+\rho_{w}(1-\alpha_{b})\right](1-\alpha_{s})+\rho_{s}\alpha_{s}$$

(9) $$\mu =\left[\mu_{b}\alpha_{b}+\mu_{w}(1-\alpha_{b})\right](1-\alpha_{s})+\mu_{s}\alpha_{s}$$

(10) $$\alpha_{b/s}=\max\left[0,\min\left(1,\frac{1}{2}+\frac{\phi_{b/s}}{h}\right)\right]$$

(11) $$\kappa =\nabla \cdot \frac{\nabla \phi_{b}}{\left|\nabla \phi_{b}\right|}$$

(12) $$H_{b}=1\;\;\mathrm{if}\;\phi_{b}>0$$

(13) $$=0\;\;\mathrm{if}\;\phi_{b}\leq 0$$

Here, $B,\tau_{e},G$ are left Cauchy-Green deformation tensor, an elastic stress tensor for neo-Hookean materials, shear elastic modulus, respectively, and α is a smoothed Heaviside function that varies linearly along the interface thickness of 1h where *h* is a grid size.

The LS functions $\phi_{b}$ and $\phi_{s}$ for tracking the bubble-water and water-tissue interfaces are advanced and reinitialized with the following equations:

(14) $$\frac{\partial \phi_{b/s}}{\partial t}+u\cdot \nabla \phi_{b/s}=0$$

(15) $$\frac{\partial \phi_{b/s}}{\partial t^{*}}=\frac{\phi_{b/s}}{\sqrt{\phi_{b/s}^{2}+h^{2}}}(1-|\nabla \phi_{b/s}|)\mathrm{if}|\;\phi_{b/s}|>h/2$$

where $t^{*}$ is an artificial time for iteratively solving Eq. (15) and $\phi_{b/s}$ are not reinitialized in the vicinity of the interface ($|\phi_{b/s}|<0.5h$) for better volume conservation.

The governing equations for air bubble, water and tissue phases are spatially discretized in a staggered grid system. Further details for spatial and temporal discretization of the governing Eqs. (4), (5), (6) and validation tests of the LS formulation for solid deformation and ultrasound-driven bubble motion have been addressed in our previous studies [31], [37].

### 2.2 Computational conditions

In this calculations, we consider a stationary air bubble and nearby viscoelastic tissue immersed in ambient water at $p_{\infty }=1\mathrm{atm}$, referring to Fig. 1, and choose the physical properties of air, water and tissue as follows: $\sigma =7.28\times 10^{-2}\;N/m$, $\mu_{w}=\mu_{s}=10^{-3}\;\mathrm{Pa}\cdot s$, $\rho_{w,\infty }=998\;\mathrm{kg}/m^{3}$, $\Pi_{w}=3.31\times 10^{8}\;\mathrm{Pa}$, $\mu_{b}=1.8\times 10^{-5}\;\mathrm{Pa}\cdot s$, and $\rho_{b,\infty }=1.2\;\mathrm{kg}/m^{3}$. A spherical microbubble with an initial radius $R_{\mathrm{bo}}$ lies on the central axis at $y=l_{o}$ in a cylindrical domain of $r⩽R_{d,e}$ and $Y_{d,s}⩽y⩽Y_{d,n}$, where $R_{d,e}=5897R_{\mathrm{bo}},Y_{d,s}=-5897R_{\mathrm{bo}}$ and $Y_{d,n}=508R_{\mathrm{bo}}$. In the cylindrical domain, uniform fine grids with $h_{\min}=0.1\;\mu m$ are chosen around the bubble with $r<19.2R_{\mathrm{bo}}$ and $|y|<19.2R_{\mathrm{bo}}$, whereas nonuniform coarse grids for the other regions.

The sinusoidal ultrasonic pulse $p_{u}+p_{\infty }$ is applied to the upper boundaries of $y=Y_{d,n}$ as follows.

(16) $$p_{u}=-A_{u}\sin(2\pi f_{u}t)\;\;\mathrm{if}\;f_{u}t<1$$

$$=0\;\;\;\;\;\;\;\;\;\;\;\;\mathrm{if}\;f_{u}t>1$$

where $A_{u}$ and $f_{u}$ are the ultrasonic pulse amplitude and frequency, respectively. In the present computations, we keep $A_{u}=0.3\mathrm{MPa},f_{u}=1\mathrm{MHz}$ and $\gamma_{s}=\gamma_{w}$ while changing $l_{o},G,\Pi_{s}$ and $\rho_{s}$. The symmetric condition is imposed at r=0 and the convective pressure condition at $y=Y_{d,s}$. It is noted that the domain sizes of $R_{d,e}$ and $Y_{d,s}$ are chosen to be nearly four times the wavelength ($a_{w}/f_{u}$) to ensure that the bubble motion is not disturbed by any reflection from the boundaries.

We select $R_{\mathrm{bo}}=1\;\mu m$ for $f_{u}=1\;\mathrm{MHz}$. This choice is reasonable, considering Dehane and Merouani’s choice of $R_{\mathrm{bo}}=2\;\mu m$ at 1 MHz for of single-bubble sonochemistry analysis [38], based on the experimental measurements for air sonoluminescing bubbles [39], and Yasui’s numerical observation of $R_{\mathrm{bo}}=0.1-3\;\mu m$ at 1 MHz for multibubble sonoluminescence [40].

## 3 Results and discussion

### 3.1 Acoustic cavitation

**Fig. 2:**
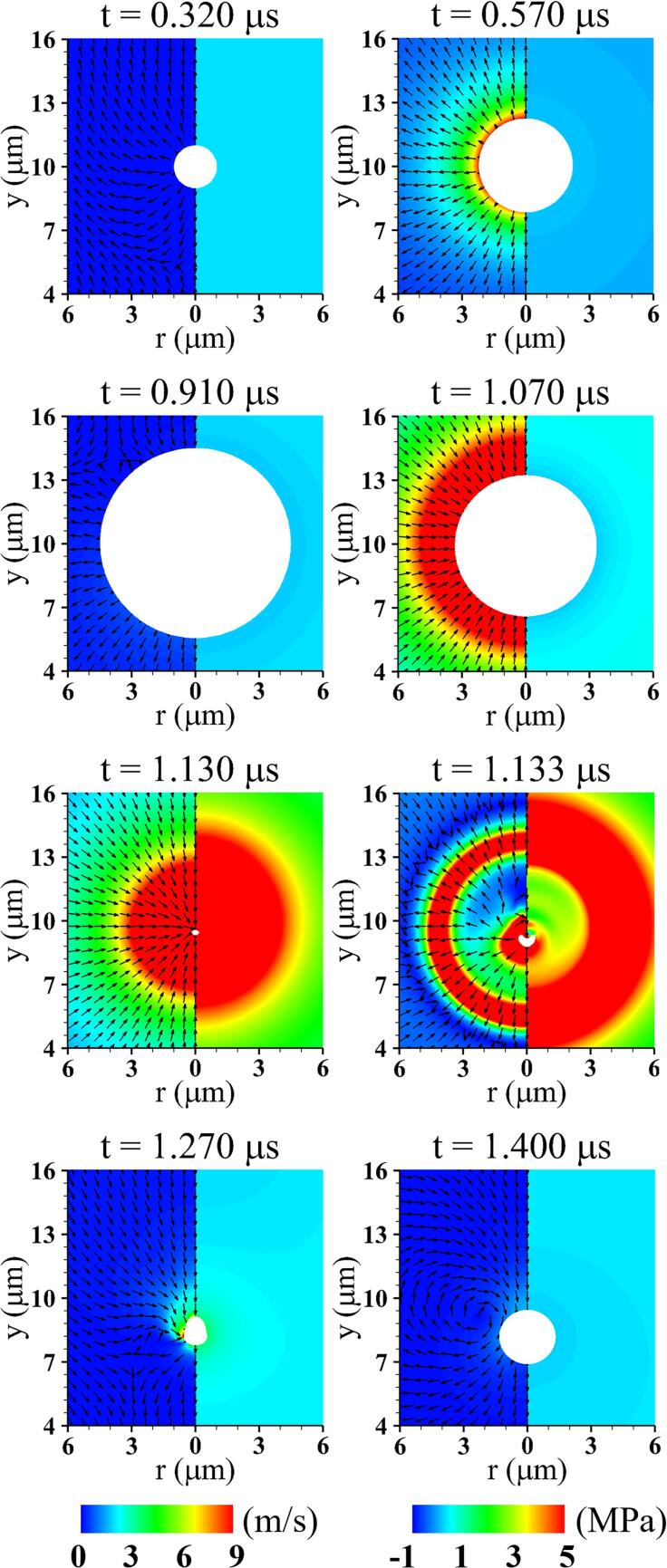
Liquid velocity field with direction vectors (left) and pressure field (right) associated with acoustic bubble motion without including nearby tissue at $A_{u}=0.3\;\mathrm{MPa},f_{u}=1\;\mathrm{MHz}$. Here, the white area represents the bubble.

Calculations are first performed for acoustic cavitation bubble without including surrounding tissue at $A_{u}=0.3\;\mathrm{MPa}$ and $f_{u}=1\;\mathrm{MHz}$. The results are presented in Fig. 2. The microbubble begins to grow at t=0.32μs when an ultrasonic pulse traveling through the ambient water at the speed of sound reaches the bubble surface. During the negative pressure pulse stage (0.32μs<t<0.82μs), the bubble grows and pushes out the surrounding liquid, forming a source flow outward from the bubble center. While the positive pressure pulse is transferred to the bubble (t>0.82μs), the bubble expands further due to the fluid inertia around its surface and then begins to contract at t=0.910μs. As the bubble shrinks rapidly, a high pressure field is created near the bubble interface, and when its volume becomes minimum, a shock wave forms, as seen at t=1.130μs. Thereafter, the bubble collapses and rebounds into a non-spherical shape at t=1.133μs, propagating a shock wave through the ambient liquid. This is coincident with previous numerical studies showing that the pressure gradient and direction of the acoustic wave determine non-spherical bubble dynamics even in the absence of surrounding walls or obstacles [41], but the asymmetric nature of the flow field is not large enough to cause liquid jet formation. After re-expansion, the bubble returns to a nearly spherical shape with subsequent oscillations and reaches an equilibrium state due to the surface tension and liquid viscous effects.

For validation of the obtained results, we consider the following Keller-Miksis equation [42] for the spherical bubble motion under a plane pressure wave:

(17) $$\left(1-\frac{\overset{˙}{R_{b}}}{C_{w}}\right)R_{b}\overset{¨}{R_{b}}+\frac{3}{2}{\overset{˙}{R_{b}}}^{2}\left(1-\frac{\overset{˙}{R_{b}}}{3C_{w}}\right)=\frac{1}{\rho_{w,\infty }}\left(1+\frac{\overset{˙}{R_{b}}}{C_{w}}+\frac{R_{b}}{C_{w}}\frac{d}{\mathrm{dt}}\right)(p_{l}+p_{u})$$

where

(18) $$p_{l}=p_{b}-2\sigma_{b}/R_{b}-4\mu {\overset{˙}{R}}_{b}/R_{b}$$

**Fig. 3:**
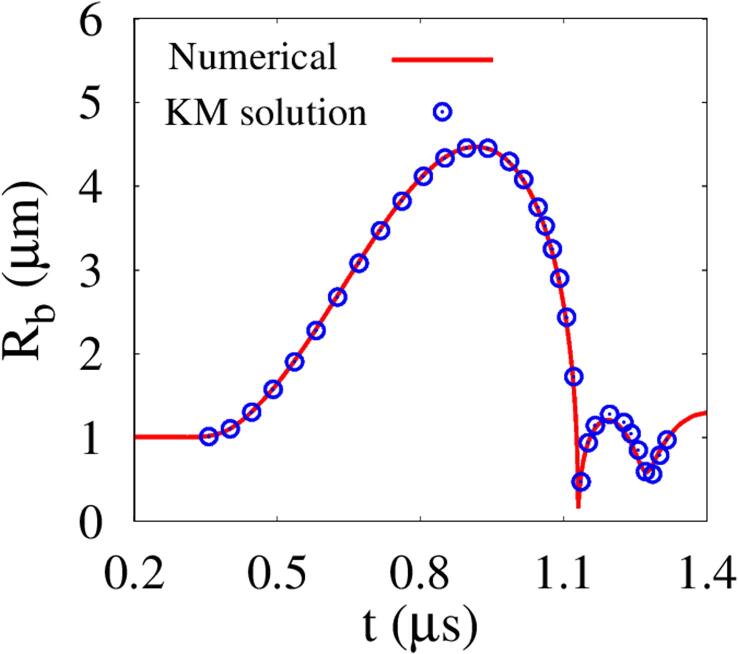
Acoustic bubble growth without including nearby tissue compared with the Keller-Miksis prediction at $A_{u}=0.3\;\mathrm{MPa},f_{u}=1\;\mathrm{MHz}$.

Here, the bubble pressure $p_{b}$ is obtained from Eq. (2) and the bubble mass balance of $\rho_{b}=\rho_{\mathrm{bo}}R_{\mathrm{bo}}^{3}/R_{b}^{3}$, and $C_{w}$ is the sound speed in liquid water. Fig. 3 compares our numerical results with the theoretical prediction from the KM Eq. (17). The volume-averaged bubble radius $R_{b}$ from the present simulation agrees well with the theoretical prediction for the growth and contraction period of the bubble. This indicates that the present numerical model is consistent with the theoretical cavitation model when the bubble-tissue distance is long.

### 3.2 Influence of bubble-tissue distance

**Fig. 4:**
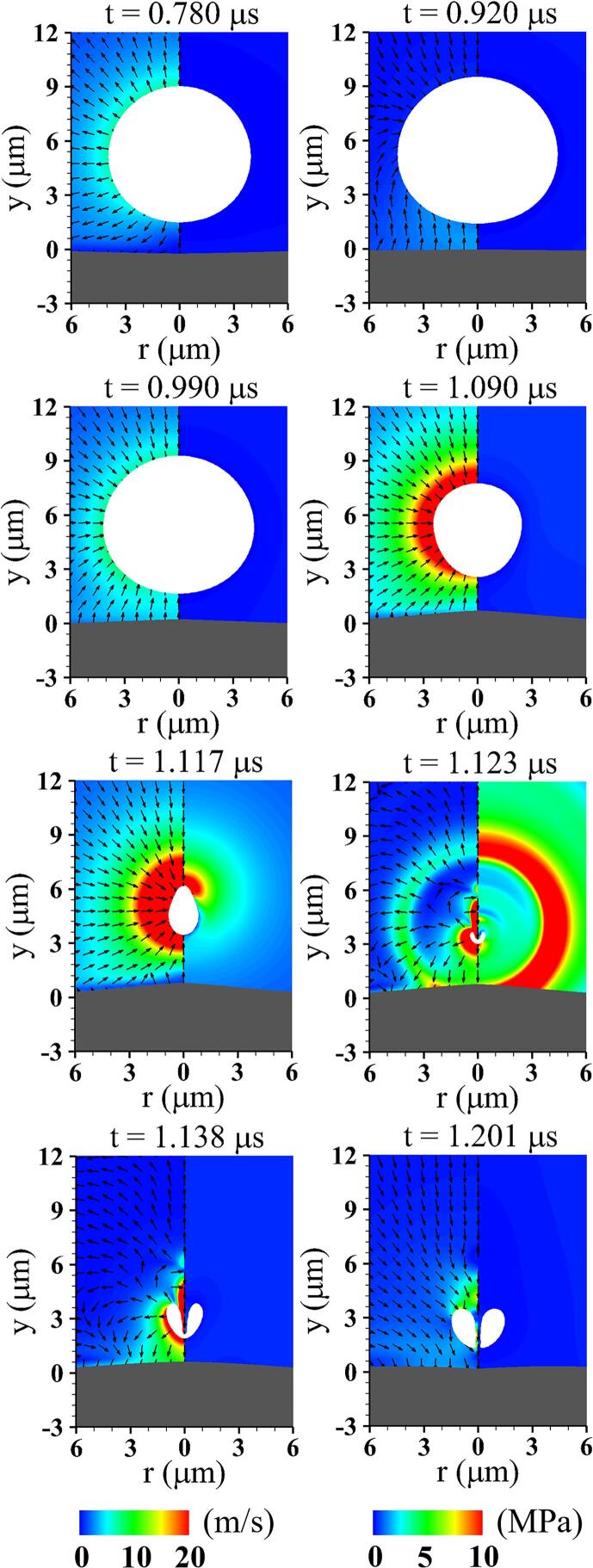
Liquid velocity field with direction vectors (left) and pressure field (right) associated with acoustic cavitation and tissue deformation at $l_{o}=5\;\mu m$ and G=1MPa. The gray and white regions represent the tissue and bubble, respectively.

Fig. 4 shows the acoustic cavitation near a viscoelastic tissue and the associated liquid flow fields at $l_{o}=5\;\mu m,G=1\;\mathrm{MPa},\Pi_{s}=\Pi_{w}$ and $\rho_{s}=\rho_{w}$. While the bubble grows due to the negative acoustic pressure, it pushes away the surrounding liquid water and nearby deformable tissue. The compressed tissue rebounds due to its elastic restoring force and pushes the surrounding liquid upward at t=0.920μs, transferring its momentum to the bottom of the bubble surface. This perturbation propagates upward from the bubble bottom along the interface, forming a relatively fast velocity field around the bubble bottom, as observed at t=0.990μs. This results in a larger interfacial curvature in the bubble bottom part compared to the top part, as seen at t=1.090μs. As the bubble shrinks, the surrounding tissue expands upward by a sink flow developed toward the bubble center, which can be confirmed from previous numerical investigation [21] based on an improved ghost fluid method. The resistance of the underlying tissue causes the upper part of the bubble to contract more rapidly than the lower part, and an annular inflow parallel to the tissue boundary induces a large curvature at the top part, as depicted at t=1.117μs. The resulting asymmetric flow field between the bubble top and bottom parts induces a liquid jet formation and downward bubble migration. The bubble is pierced by a downward liquid jet at t=1.201μs as the bubble re-expands, but is not strong enough to cause significant deformation of the tissue.

**Fig. 5:**
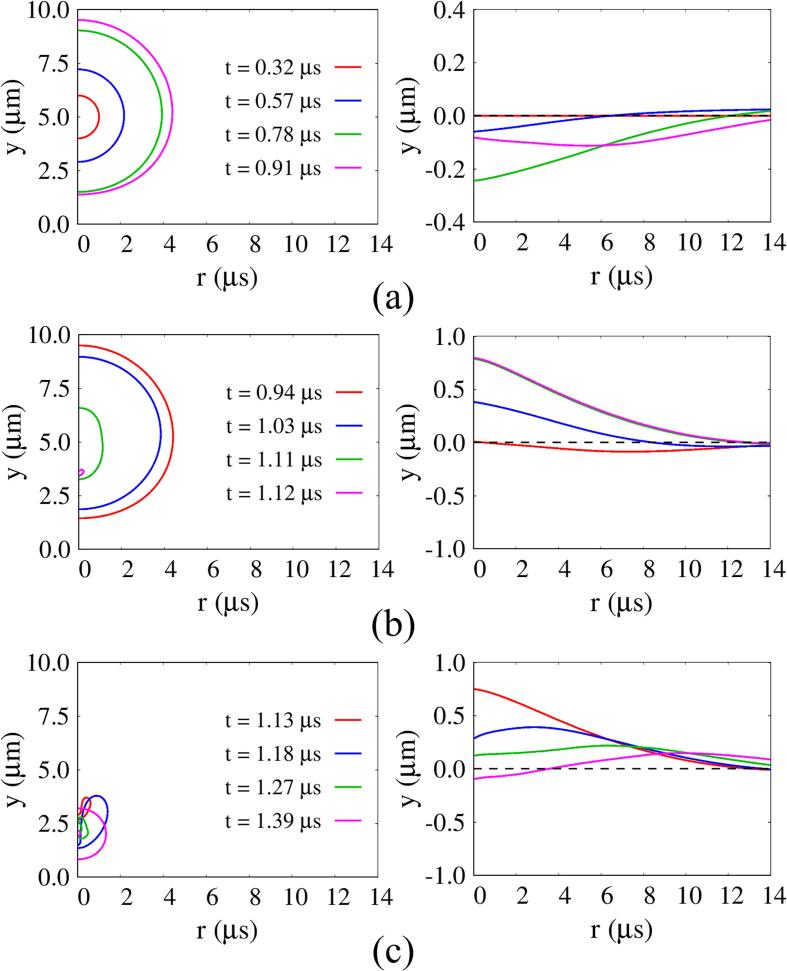
Detailed bubble motion and tissue deformation during three different periods at $l_{o}=5\;\mu m$ and G=1MPa: (a) bubble growth, (b) contraction, and (c) rebound.

Fig. 5 presents details of the bubble and tissue interfaces in Fig. 4 during the first period of bubble motion. While the bubble initially expands (t<0.91μs), it pushes out the surrounding tissue, causing the tissue to undergo compressive deformation until t=0.78μs, as presented in Fig. 5(a). As the bubble expansion rate decreases, the elastic restoring force in the compressed tissue exceeds the liquid momentum transferred to the tissue surface, raising the tissue position on the central axis by 30% of the maximum compressive deformation at t=0.91μs. During the bubble contraction phase (0.91μs<t<1.12μs), the tissue position returns to y=0 at t=0.94μs and is gradually attracted toward the bubble. The bubble sides contract rapidly, which induces an elongated shape of the bubble along the vertical axis. Thereafter, as the bubble rapidly collapses with downward migration, the tissue expands to reach its highest position at t=1.12μs. During the bubble rebound phase (t>1.12μs), the downward movement of the re-expanding bubble once again lowers the tissue surface below y=0 with rapid compressive deformation.

**Fig. 6:**
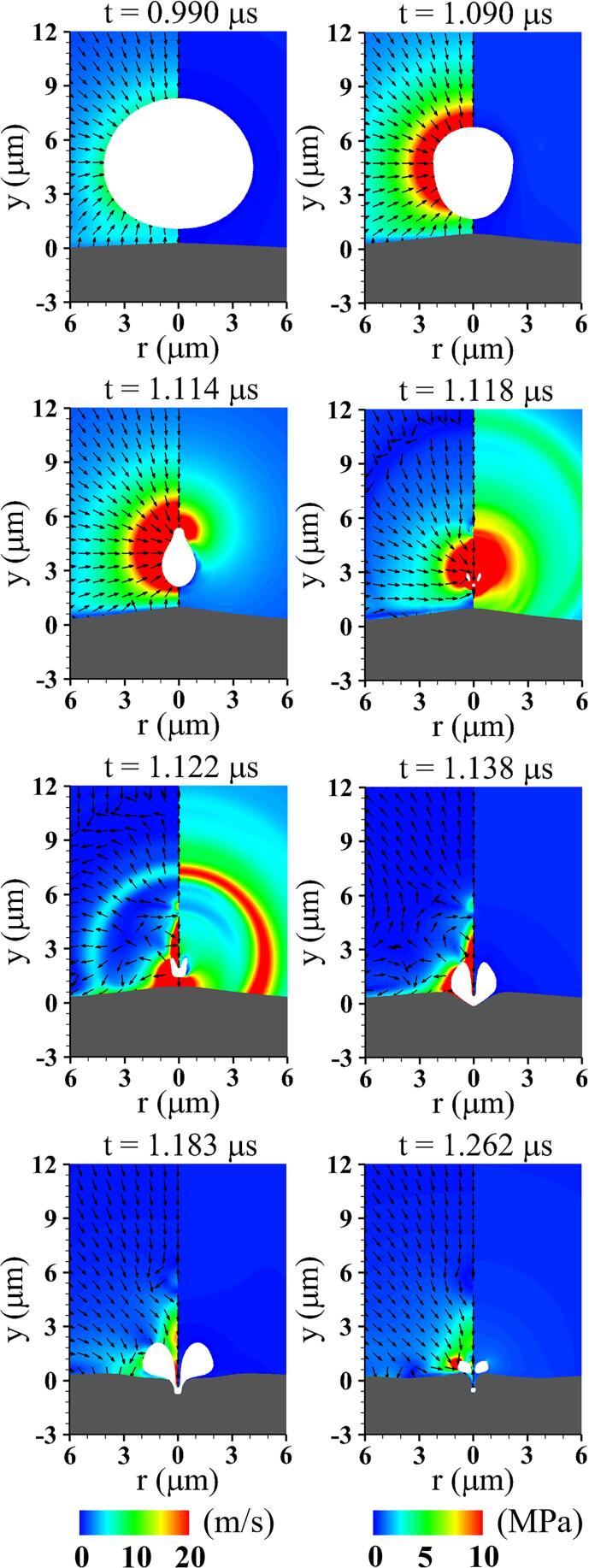
Liquid velocity field with direction vectors (left) and pressure field (right) associated with acoustic cavitation and tissue deformation at $l_{o}=4\;\mu m$ and G=1MPa.

When $l_{o}$ decreases to 4μm, the resulting flow fields are plotted in Fig. 6. As the liquid between the bubble and tissue interfaces is released during the bubble growth period and a thin liquid layer develops beneath the bubble, the bubble-tissue interaction becomes stronger, resulting in an inverted cone-shaped bubble at t=1.090μs, which was also observed in the previous experimental study [17]. Due to the flow resistance of nearby tissue, a highly asymmetric velocity field develops along the y-axis and the upper part of the bubble surface rapidly contracts compared to the lower part. At t=1.114μs, the annular inflow towards the central axis impinges on the top of the bubble surface, forming an extreme interfacial curvature, which causes the liquid to be squeezed into the bubble in the form of a strong vertical jet. After the bubble collapse (t>1.118μs), the bubble migrates rapidly downward and almost touches the tissue surface, resulting in compressive tissue deformation accompanied by bubble re-expansion. As the bubble recontracts and the tissue undergoes expansive deformation toward the nearby bubble, narrowing the pore entrance, a small portion of the bubble becomes trapped inside the tissue, causing long-term tissue deformation, as depicted at t=1.262μs.

**Fig. 7:**
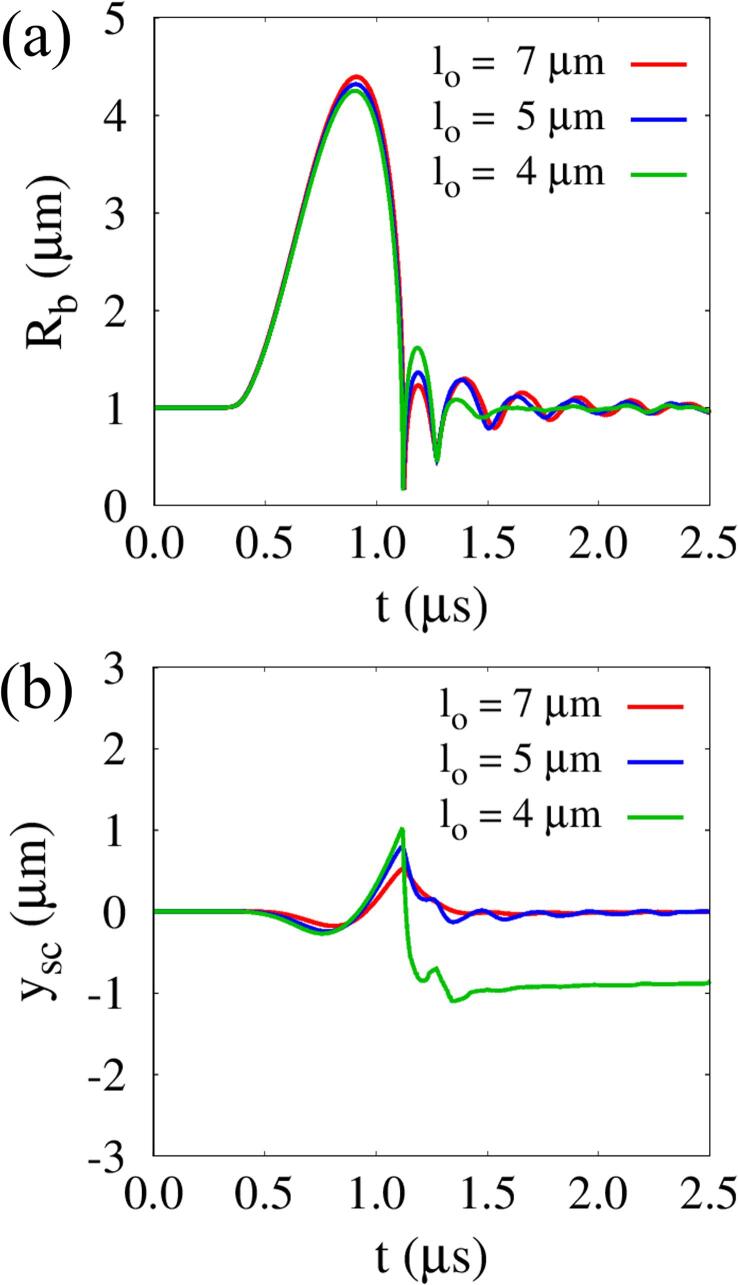
Influence of $l_{o}$ on acoustic cavitation bubble and tissue behavior at $G=1\;\mathrm{MPa},\Pi_{s}=\Pi_{w}$ and $\rho_{s}=\rho_{w}$: (a) temporal change of $R_{b}$ and (b) tissue deformation $y_{\mathrm{sc}}$ at the central axis (r=0).

Fig. 7 shows the influence of $l_{o}$ on $R_{b}$ and tissue surface location $y_{\mathrm{sc}}$ at the central axis (r=0) for $G=1\;\mathrm{MPa},\Pi_{s}=\Pi_{w}$ and $\rho_{s}=\rho_{w}$. When an ultrasonic pulse traveling through the liquid water reaches the bubble at t=0.32μs, $R_{b}$ begins to increase from $R_{\mathrm{bo}}=1\;\mu m$. During the ultrasound pulse period, bubble contraction proceeds faster than bubble expansion, which can be observed in the previous cavitation models [10], [14]. This is because the ultrasonic pulsed positive pressure is applied to a larger bubble area during expansion. As the initial bubble-tissue distance decreases from $l_{o}=7\;\mu m$ to $l_{o}=4\;\mu m$, the maximum bubble radius $R_{b,\max}$ decreases slightly by about 3.3%. During the first cycle of bubble growth and contraction, the tissue that resists the surrounding fluid flow further impedes bubble motion as $l_{o}$ decreases. Thereafter, the bubble at $l_{o}=4\;\mu m$ re-expands faster than the other cases, and the second maximum bubble radius is about 34% larger than that at $l_{o}=7\;\mu m$. This can be explained by the fact that the liquid kinetic energy loss by shock wave emission is relatively small at $l_{o}=4\;\mu m$, which is consistent with the previous statements that bubble collapse near a wall causes less energy release and larger bubble re-expansion than farther from the wall [43]. The effect of $l_{o}$ on tissue deformation compared to bubble motion is more pronounced at $l_{0}=4\;\mu m$, as seen in Fig. 7(b). The tissue surface reaches the first minimum location of $y_{\mathrm{sc},\min1}=-0.27\;\mu m$ at t=0.76μs and the maximum location of $y_{\mathrm{sc},\max}=1.01\;\mu m$ at t=1.12μs, whose magnitudes are about 52% and 92% larger than those for $l_{o}=7\;\mu m$, respectively. After bubble collapse, the tissue is rapidly compressed to reach the second minimum location of $y_{\mathrm{sc},\min2}=-1.10\;\mu m$ and then undergoes long-lasting deformation. This is because the tissue surface is not restored due to the bubble portion trapped inside the tissue, as depicted in Fig. 5.

### 3.3 Influence of shear modulus

**Fig. 8:**
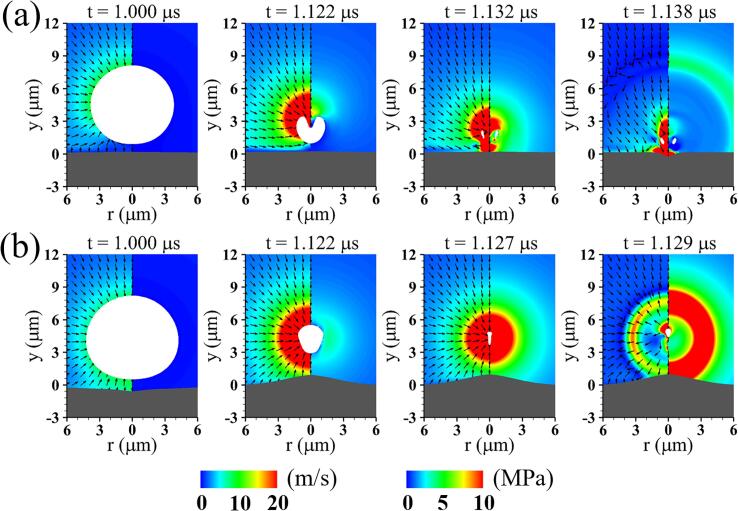
Liquid velocity field with direction vectors (left) and pressure field (right) associated with acoustic cavitation and tissue deformation at $l_{o}=4\;\mu m,\Pi_{s}=\Pi_{w},\rho_{s}=\rho_{w}$ and two different shear moduli: (a) G=10MPa and (b) G=0.1MPa.

Fig. 8 presents the liquid flow fields associated with acoustic cavitation and tissue deformation for two different shear moduli, G=10MPa and G=0.1MPa. The tissue with G=10MPa behaves like a rigid wall and little liquid momentum due to tissue rebound is transmitted to the bubble, as depicted at t=1.000μs in Fig. 8(a). The stiff tissue hinders the fluid flow around the lower part of the bubble, causing non-spherical bubble contraction at t=1.122μs, which was observed in the pervious numerical computation of near-wall bubble dynamics [43]. The fast liquid inflow on the bubble top surface induces rapid downward bubble migration and strong liquid jet formation. The liquid jet tip close to the tissue transfers dense liquid momentum to the adjacent tissue surface, resulting in rapid compressive deformation at t=1.138μs. Compared to the case of G=10MPa, the more flexible tissue with G=0.1MPa has larger rebound and causes faster contraction of the bottom bubble surface than the top surface, as seen in Fig. 8(b). This results in a violent liquid annular inflow parallel to the tissue surface and pronounced inverted cone-like bubble shape at t=1.122μs. The annular flow impinges the bubble sides and produces a mushroom-shaped bubble characterized by depressions of the lateral sides, which can be observed in the previous experimental and numerical researches for bubble-tissue interactions [34], [44]. At t=1.127μs, the bubble collapses into a thin, elongated shape and is driven away from the tissue surface by the asymmetric velocity field along the vertical axis and the large curvature of the bubble bottom. This indicates that the location of bubble collapse and liquid jet formation is influenced by tissue elasticity as well as bubble-tissue distance.

**Fig. 9:**
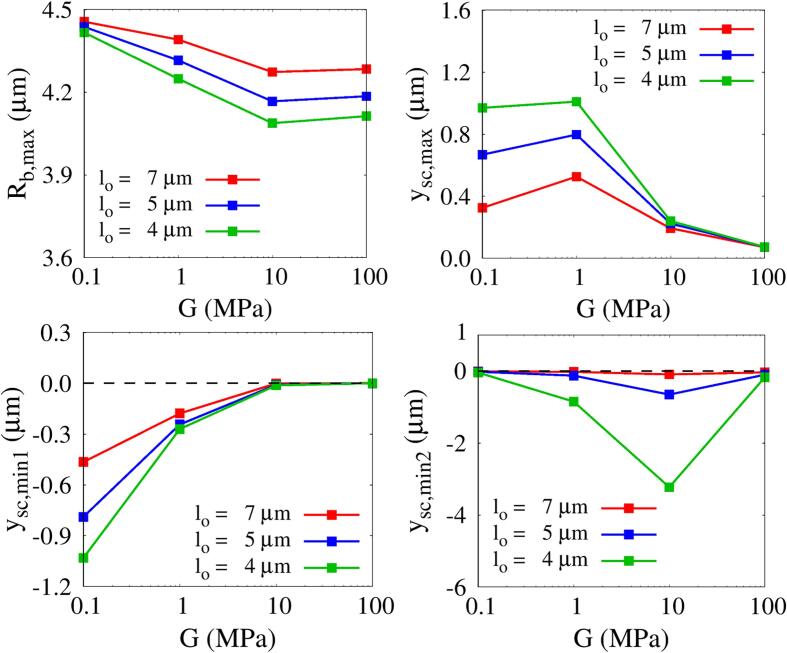
Combined effects of $l_{o}$ and *G* on bubble growth and tissue deformation at $\Pi_{s}=\Pi_{w}$ and $\rho_{s}=\rho_{w}$.

Fig. 9 shows the integrated influences of $l_{o}$ and *G* on the bubble maximum radius $R_{b,\max}$ and tissue deformation while keeping $\Pi_{s}=\Pi_{w}$ and $\rho_{s}=\rho_{w}$. As $l_{o}$ decreases, $R_{b,\max}$ decreases while the tissue deformations $\left|y_{\mathrm{sc},\min1}|,|y_{\mathrm{sc},\min2}\right|$ and $y_{\mathrm{sc},\max}$ increase. The effect of *G* is more pronounced in the small $l_{o}$ range, where the liquid momentum is transferred more directly. For $l_{o}=4\;\mu m$, $R_{b,\max}$ decreases monotonically as the shear modulus increases from G=0.1MPa. Similarly, during the bubble growth phase, the first compressive tissue deformation $\left|y_{\mathrm{sc},\min1}\right|$ gradually decreases with increasing *G*. However, $R_{b,\max}$ and $y_{\mathrm{sc},\min1}$ do not vary for G>10MPa due to the large tissue resistance. On the other hand, $y_{\mathrm{sc},\max}$ does not monotonically increase or decrease with *G* and is largest near G=1MPa. This can be explained by the competing effects of tissue repulsion due to the elastic restoring force and tissue attraction toward the center of the contracting bubble. The second compressive tissue deformation $\left|y_{\mathrm{sc},\min2}\right|$ that occurs after bubble collapse is found to be clearly maximized near G=10MPa, which is the condition for strong liquid jet formation and resulting tissue compressive deformation. The deformation $\left|y_{\mathrm{sc},\min2}\right|$ decreases rapidly beyond the point of G=10MPa. This indicates that tissue shear modulus is a key parameter for tissue deformation large enough to cause perforation.

### 3.4 Effect of tissue bulk modulus

**Fig. 10:**
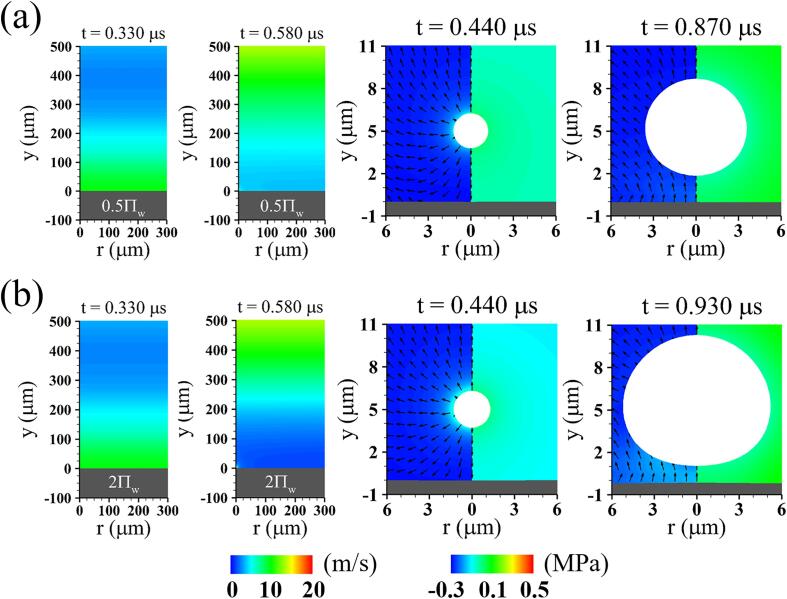
Instantaneous liquid pressure field without including bubble motion (two figures on the left) and instantaneous flow fields associated with acoustic cavitation and tissue deformation at $l_{o}=5\;\mu m,G=1\;\mathrm{MPa},\rho_{s}=\rho_{w}$ and different bulk moduli: (a) $\Pi_{s}=0.5\Pi_{w}$ and (b) $\Pi_{s}=2\Pi_{w}$.

Fig. 10 describes the effect of tissue bulk modulus ${\left(\gamma \Pi \right)}_{s}$ on the liquid pressure field and bubble growth at $l_{o}=5\;\mu m,G=1\;\mathrm{MPa}$ and $\rho_{s}=\rho_{w}$. It is noted that the acoustic impedance $I_{s}=\rho_{s}C_{s}$, where $C_{s}$ is the speed of sound through the tissue, is approximated as $I_{s}=\sqrt{{\left(\rho \gamma \Pi \right)}_{s}}$ from Eq. (1). The ultrasonic wave traveling through the liquid water reaches the underlying tissue with $\Pi_{s}=0.5\Pi_{w}$ at $t=Y_{d,n}/C_{w}=0.33\;\mu s$, as seen in Fig. 10(a). It is partially reflected by the tissue, which has a smaller acoustic impedance ($I_{s}=\sqrt{0.5}I_{w}$) than the ambient water, corresponding to a negative reflection coefficient of $(I_{s}-I_{w})/(I_{s}+I_{w})=-0.17$. Since the incident and reflected waves are out of phase, the superimposed liquid pressure is less negative around the bubble, which causes the bubble to reach a smaller maximum size at t=0.870μs compared to the case of $\Pi_{s}=\Pi_{w}$. On the other hand, when the tissue has a larger acoustic impedance of $\Pi_{s}=2\Pi_{w}$, the superimposed liquid pressure is more negative around the bubble because the reflected wave is in phase with the incident wave. This results in faster bubble growth and larger bubble size at t=0.930μs.

**Fig. 11:**
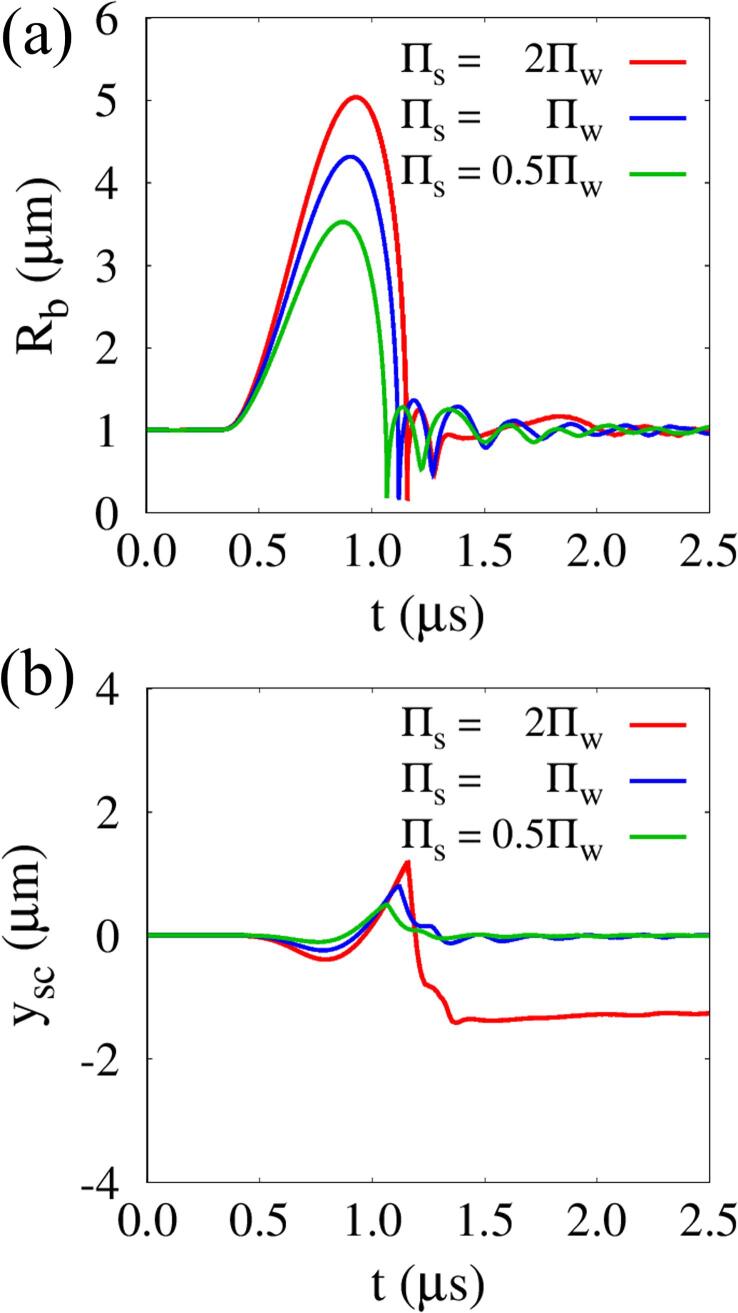
Effect of tissue bulk modulus on acoustic cavitation bubble and tissue behavior at $l_{o}=5\;\mu m,G=1\;\mathrm{MPa}$, and $\rho_{s}=\rho_{w}$: (a) temporal change of $R_{b}$ and (b) tissue deformation $y_{\mathrm{sc}}$ at the central axis (r=0).

The effect of $\Pi_{s}$ on the temporal behavior of $R_{b}$ and $y_{\mathrm{sc}}$ is plotted in Fig. 11. For $\Pi_{s}=2\Pi_{w}$, while the ultrasonic negative wave overlaps with the reflected wave around the tissue surface, the bubble grows rapidly and reaches $R_{b,\max}=5.04\;\mu m$ at t=0.930μs, which is 16.7% larger than for $\Pi_{s}=\Pi_{w}$, as seen in Fig. 11(a). After the bubble expansion stage, the bubble begins to shrink and collapses rapidly, and the ratios of the bubble collapse time $\Delta t_{\mathrm{col}}$ from its maximum radius compared to that for $\Pi_{s}=\Pi_{w}$ are estimated as 0.92 and 1.06 for $\Pi_{s}=0.5\Pi_{w}$ and $2\Pi_{w}$, respectively. This can be qualitatively comparable to the ratios, 0.94 and 1.04, of the Rayleigh collapse time [43] proportional to $R_{b,\max}/\sqrt{|p_{s,\max}-p_{\infty }|/\rho_{w}}$, with slight deviations due to the presence of surrounding tissue. Here, $p_{s,\max}$ is the pressure at $\left(r,y)=(R_{d,e},0\right)$ when the maximum positive pulse reaches to the tissue surface. The changes in tissue bulk modulus also affect the temporal behavior of the tissue. During the initial bubble expansion and contraction period, the first compressive and expansive tissue deformations $y_{\mathrm{sc},\min1}$ and $y_{\mathrm{sc},\max}$ become more pronounced as $\Pi_{s}$ increases. For $\Pi_{s}=2\Pi_{w}$, the tissue deformed to $y_{\mathrm{sc},\min1}=-0.39\mu m$ rapidly expands, reaching $y_{\mathrm{sc},\max}=1.18\;\mu m$, which is 47.5% larger than that for $\Pi_{s}=\Pi_{w}$. After bubble collapse, the tissue is rapidly compressed and perforated, arriving at $y_{\mathrm{sc},\min2}=-1.42\;\mu m$. This indicates that acoustic cavitation near a tissue with larger tissue bulk modulus, leading very asymmetric velocity fields, is more likely to cause larger tissue deformation with perforation.

**Fig. 12:**
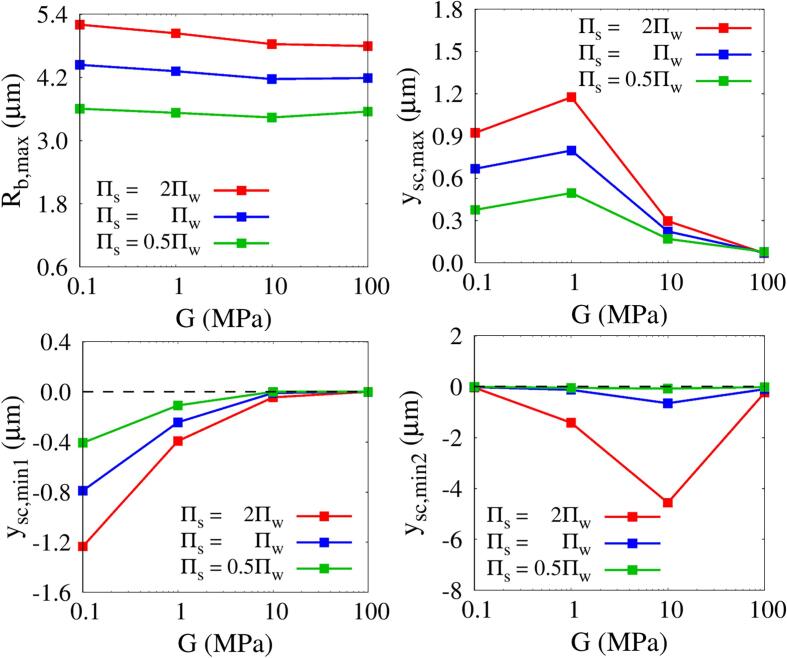
Combined effects of tissue bulk modulus and shear modulus on bubble growth and tissue deformation at $l_{o}=5\;\mu m$ and $\rho_{s}=\rho_{w}$.

Fig. 12 shows the combined effects of $\Pi_{s}$ and *G* on $R_{b,\max}$ and tissue deformation at $l_{o}=5\;\mu m$ and $\rho_{s}=\rho_{w}$. As the acoustic impedance of the tissue increases with $\Pi_{s}$, $R_{b,\max},|y_{\mathrm{sc},\min1}|,|y_{\mathrm{sc},\min2}|$ and $y_{\mathrm{sc},\max}$ all increase over almost the entire range of *G*. Their dependence on *G* for various $\Pi_{s}\prime s$ is almost similar to that on *G* for various $l_{o}\prime s$, except for $R_{b,\max}$ in the range of G<1MPa, as seen in Fig. 9. Regardless of the value of $\Pi_{s}$, the maximum expansive deformation $y_{\mathrm{sc},\max}$ of the tissue occurs at G=1MPa and the maximum compressive deformation $\left|y_{\mathrm{sc},\min2}\right|$ occurs at G=10MPa. The deformation $\left|y_{\mathrm{sc},\min2}\right|$ is 4.57μm at $\Pi_{s}=2\Pi_{w}$ and $l_{o}=5\;\mu m$, which is 41% larger than $\left|y_{\mathrm{sc},\min2}\right|$ at $\Pi_{s}=\Pi_{w}$ and $l_{o}=4\;\mu m$ in Fig. 9. This is because the intensity of liquid jet formed by the non-spherical and rapid contraction of the bubble becomes stronger as $R_{b,\max}$ increases, which is similarly shown in the numerical observation that the jet velocity is proportional to the bubble expansion size ratio [45].

### 3.5 Effect of tissue density

**Fig. 13:**
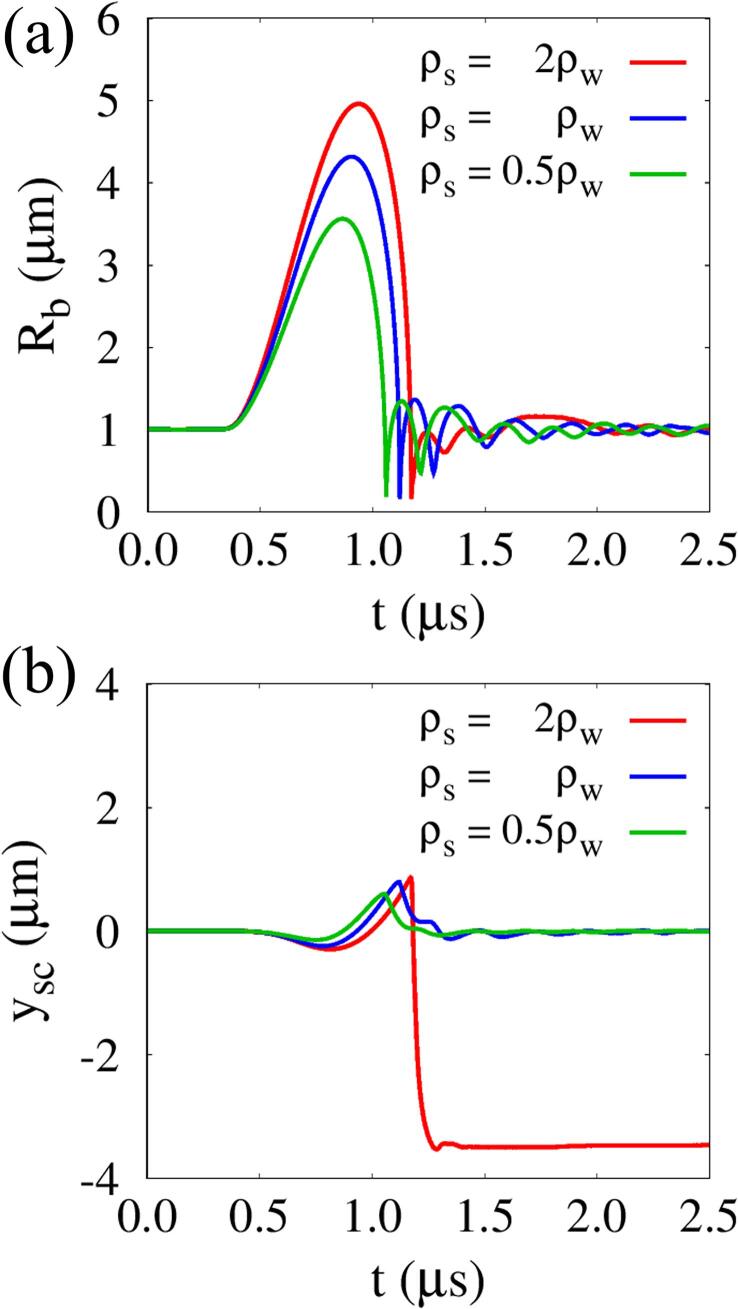
Effect of tissue density on acoustic cavitation bubble and tissue deformation at $l_{o}=5\;\mu m$ ,G=1MPa, and $\Pi_{s}=\Pi_{w}$: (a) temporal change of $R_{b}$ and (b) tissue deformation $y_{\mathrm{sc}}$ at the central axis (r=0).

Fig. 13 presents the effect of tissue density $\rho_{s}$ on acoustic cavitation bubble growth and tissue deformation at $l_{o}=5\;\mu m,G=1\;\mathrm{MPa}$ and $\Pi_{s}=\Pi_{w}$. During the first period of bubble growth and collapse, the temporal variations of $R_{b}$ for $\rho_{s}=2\rho_{w},\rho_{w}$ and $0.5\rho_{w}$ in Fig. 13(a) are similar to those for $\Pi_{s}=2\Pi_{w},\Pi_{w}$ and $0.5\Pi_{w}$ in Fig. 10(a), respectively. This can be expected form the fact that the acoustic impedance $I_{s}=\sqrt{{\left(\rho \gamma \Pi \right)}_{s}}$ is the same for each case. However, the acoustic cavitation bubble-induced tissue deformation pattern is affected differently by $\rho_{s}$ compared to $\Pi_{s}$, as seen in Fig. 13(b). As the tissue inertia increases with $\rho_{s}$, the tissue responds more slowly to the surrounding bubble motion. The tissue with $\rho_{s}=2\rho_{w}$ is compressed to reach $|y_{\mathrm{sc},\min1}|=0.29\;\mu m$, which is 26% smaller than for $\Pi_{s}=2\Pi_{w}$. Thereafter, the tissue surface rises due to tissue elasticity and sink flow toward the bubble center and reaches $y_{\mathrm{sc},\max}=0.86\;\mu m$, which is 27% lower than for $\Pi_{s}=2\Pi_{w}$. However, during bubble collapse and re-expansion, the tissue undergoes violent compressive deformation by the strong liquid jet to $|y_{\mathrm{sc},\min2}|=3.53\;\mu m$, which is 2.48 times lager than for $\Pi_{s}=2\Pi_{w}$. This implies that tissue density is a more important factor in determining strong liquid jet formation and large tissue deformation than tissue bulk modulus.

**Fig. 14:**
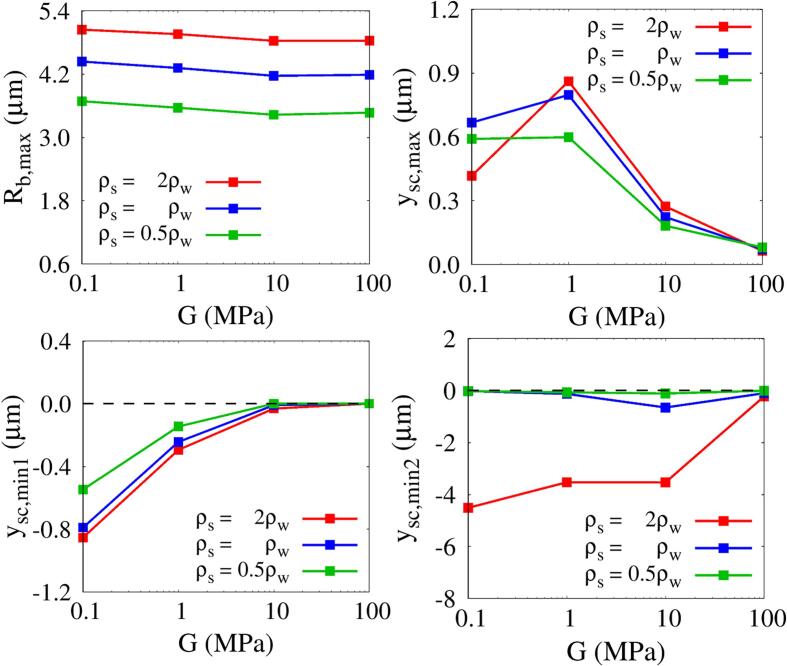
Combined effects of tissue density and shear modulus on bubble growth and tissue deformation at $l_{o}=5\;\mu m$ and $\Pi_{s}=\Pi_{w}$.

The combined effects of $\rho_{s}$ and *G* on $R_{b,\max}$ and tissue deformation at $l_{o}=5\mu m$ and $\Pi_{s}=\Pi_{w}$ are plotted in Fig. 14. As the acoustic impedance of the tissue increases with $\rho_{s}$, $R_{b,\max}$ increases over the entire range of *G*. Its dependence on $\rho_{s}$ and *G* is very similar to that on $\Pi_{s}$ and *G*. For $\rho_{s}=2\rho_{w}$, during the initial bubble growth period, the tissue deforms less due to its significant inertia, so $\left|y_{\mathrm{sc},\min1}\right|$ decreases less from that for $\rho_{s}=\rho_{w}$ than for $\Pi_{s}=2\Pi_{w}$. After the first tissue compression period, the expansive tissue deformation $y_{\mathrm{sc},\max}$ increases with $\rho_{s}$ over a wide range of *G*. However, in the less elastic range near G=0.1MPa, $y_{\mathrm{sc},\max}$ is 0.42μm for $\rho_{s}=2\rho_{w}$, which is smaller than the other cases for $\rho_{s}=\rho_{w}$ and $0.5\rho_{w}$. The shear modulus that maximizes $y_{\mathrm{sc},\max}$ is close to G=1MPa and the shear modulus point that maximizes the compressive tissue deformation $\left|y_{\mathrm{sc},\min2}\right|$ at $\rho_{s}=\rho_{w}$ is close to G=10MPa, as when varying $\Pi_{s}$. However, when the tissue density increases to $\rho_{s}=2\rho_{w}$, $\left|y_{\mathrm{sc},\min2}\right|$ has large values in a wide range of *G*, except near G=100MPa, where the liquid jet cannot penetrate the stiff tissue. This indicates that the tissue density is also an important parameter determining strong liquid jet formation and large tissue deformation with perforation.

## 4 Conclusions

Acoustic cavitation of a pre-existing microbubble near a viscoelastic tissue was investigated by extending our level-set method for bubble dynamics under ultrasonic pulse conditions to include the viscoelastic and compressible properties of the nearby tissue. Our numerical model was validated through comparison with the predictions from the Keller-Miksis equation including the liquid compressibility and ultrasonic conditions of plane pressure wave. We demonstrated various bubble-tissue interactions, including inverted cone-shape bubbles, bubble migration, liquid jet formation, compressive and expansive tissue deformation, and tissue perforation. The bubble was observed to grow larger with increasing tissue bulk modulus and density, and it decreases as the initial bubble-tissue distance decreases below a certain point. The maximum expansive and compressive tissue deformation generally increases with decreasing initial bubble-tissue distance and with increasing tissue bulk modulus and density, except in the case of high-density, weakly elastic tissues ($\rho_{s}=2\rho_{w}$ and G=0.1MPa). The tissue shear modulus conditions that maximize expansive and compressive tissue deformation are close to G=1MPa and G=10MPa, respectively, unless the tissue density is very large ($\rho_{s}<2\rho_{w}$). This occurs due to the competing effects of tissue elasticity and bubble-induced flow. The present numerical simulations demonstrated that initial bubble-tissue distance and tissue properties including shear modulus, bulk modulus and density are important factors in determining strong liquid jet formation and tissue deformation large enough to cause perforation.

## CRediT authorship contribution statement

**Jaesung Park:** Writing – original draft, Conceptualization, Software. **Gihun Son:** Writing – review & editing, Conceptualization, Methodology, Supervision.

## Declaration of competing interest

The authors declare that they have no known competing financial interests or personal relationships that could have appeared to influence the work reported in this paper.
